# A new human challenge model for testing heat-stable toxin-based vaccine candidates for enterotoxigenic *Escherichia coli* diarrhea – dose optimization, clinical outcomes, and CD4+ T cell responses

**DOI:** 10.1371/journal.pntd.0007823

**Published:** 2019-10-30

**Authors:** Sunniva Todnem Sakkestad, Hans Steinsland, Steinar Skrede, Kristine Lillebø, Dag Harald Skutlaberg, Anne Berit Guttormsen, Anton Zavialov, Sari Paavilainen, Hanne Søyland, Marianne Sævik, Astrid Rykkje Heien, Marit Gjerde Tellevik, Eileen Barry, Nina Langeland, Halvor Sommerfelt, Kurt Hanevik

**Affiliations:** 1 Department of Clinical Science, Faculty of Medicine, University of Bergen, Bergen, Norway; 2 Centre for Intervention Science in Maternal and Child Health (CISMAC), Centre for International Health, Department of Global Public Health and Primary Care, Faculty of Medicine, University of Bergen, Bergen, Norway; 3 Department of Biomedicine, Faculty of Medicine, University of Bergen, Bergen, Norway; 4 Division for Infectious Diseases, Department of Medicine, Haukeland University Hospital, Bergen, Norway; 5 Department of Microbiology, Haukeland University Hospital, Bergen, Norway; 6 Department of Anesthesia and Intensive Care, Haukeland University Hospital, Bergen, Norway; 7 Joint Biotechnology Laboratory, Department of Chemistry, University of Turku, Turku, Finland; 8 Norwegian National Advisory Unit on Tropical Infectious Diseases, Department of Medicine, Haukeland University Hospital, Bergen, Norway; 9 Center for Vaccine Development and Global Health, University of Maryland School of Medicine, Baltimore, United States of America; 10 Department of Medicine, Haraldsplass Deaconess Hospital, Bergen, Norway; 11 Norwegian Institute of Public Health, Ministry of Health and Care Services, Oslo, Norway; University of Texas Medical Branch, UNITED STATES

## Abstract

Enterotoxigenic *Escherichia coli* (ETEC) are a common cause of diarrheal illness in young children and travelers. There is yet no licensed broadly protective vaccine against ETEC. One promising vaccine development strategy is to target strains expressing the heat-stable toxin (ST), particularly the human ST (STh), since infections with these strains are among the leading causes of diarrhea in children in low-and-middle income countries. A human challenge model based on an STh-only ETEC strain will be useful to evaluate the protective efficacy of new ST-based vaccine candidates. To develop this model, we experimentally infected 21 healthy adult volunteers with the epidemiologically relevant STh-only ETEC strain TW10722, identified a suitable dose, assessed safety, and characterized clinical outcomes and immune responses caused by the infection. Doses of 1×10^10^ colony-forming units (CFU) of TW10722 gave a suitable attack risk of 67% for moderate or severe diarrhea and an overall diarrhea attack risk of 78%. Non-diarrheal symptoms were mostly mild or moderate, and there were no serious adverse events. During the first month after ingesting the challenge strain, we measured significant increases in both activated CD4+ T cells and levels of serum IgG and IgA antibodies targeting coli surface antigen 5 (CS5) and 6 (CS6), as well as the *E*. *coli* mucinase YghJ. The CS5-specific CD4+ T cell and antibody responses were still significantly elevated one year after experimental infection. In conclusion, we have developed a safe STh-only ETEC-based human challenge model which can be efficiently used in Phase 2B trials to evaluate the protective efficacy of new ST-based vaccine candidates.

**Trial registration:**

ClinicalTrials.gov ClinicalTrials.gov, Project ID: NCT02870751

## Author summary

Enterotoxigenic *Escherichia coli* (ETEC) is a common cause of diarrheal illness in young children living in low- and middle-income countries and in travelers to these countries. Several ETEC vaccine candidates are currently being developed, but so far, no broadly protective vaccines have been licensed. Since most moderate and severe ETEC diarrheal episodes are caused by strains that express the heat-stable enterotoxin (ST), ST represents a promising vaccine target. Here we present a human challenge model that can be used to estimate the protective efficacy of ST-based vaccine candidates in clinical vaccine trials. The model is based on the epidemiologically relevant ST-only ETEC strain TW10722, which we show is safe to ingest by volunteers and readily induce diarrhea.

## Introduction

Enterotoxigenic *Escherichia coli* (ETEC) are among the most important causes of diarrhea in low-and-middle income countries (LMICs) and of travelers’ diarrhea [1, 2]. ETEC are responsible for some 75 million diarrheal episodes and an estimated 50,000 deaths annually [1], mostly in children less than 5 years of age. This is an age where enteric infections may also cause severe sequelae such as malnutrition and impaired cognitive development [3, 4]. There is currently no licensed broadly protective vaccine against ETEC, although several candidates have reached different stages of pre-clinical and clinical testing [5], with one candidate currently in phase I and II vaccine trials [6]. Human ETEC secrete one or two types of enterotoxins called the heat-stable toxin (ST) and the heat-labile toxin (LT), both of which can induce diarrhea by binding to receptors in the small intestinal epithelium and trigger secretion of salts and fluid into the gut lumen [7]. In contrast to the large and immunogenic LT, ST is small and non-immunogenic and is found in two close to identical variants called porcine ST (STp, a.k.a. STaI or pSTa) and human ST (STh, a.k.a. STaII or hSTa) [8]. While strains producing STh only appear to cause diarrhea in humans, STp-producing strains are also often associated with diarrheal illnesses in newborn piglets and calves [9].

ETEC that express ST (with or without LT) is an important cause of moderate-to-severe diarrhea among young LMIC children. Furthermore, STh-producing strains are epidemiologically more important than STp-producing strains [10, 11]. Development of an efficient vaccine targeting diarrhea-inducing ST-ETEC strains is of great global health interest, and recently, important obstacles to produce safe and immunogenic ST-based vaccines have been overcome [12–14], partly by coupling otherwise non-immunogenic ST molecules to larger immunogens [8]. Although no ST-based vaccine candidates have reached clinical evaluation, there is now a need to prepare for these trials by developing a human challenge model that can be used to test them in Phase 2B (vaccine challenge) trials. Such a human challenge model should be based on a wild-type ETEC strain that produces STh, but not LT, since diarrhea induced by the latter would obscure protection conferred by immunity to ST, which would lead to an underestimation of vaccine-induced protection. Another important application of a human STh-only ETEC challenge model would be to evaluate the effect of LT-based adjuvants, such as the double mutant LT (dmLT) [15]. Specifically, if an LT-expressing challenge strain was used to test vaccine candidates using LT-based adjuvants, antibodies elicited by this adjuvant would potentially contribute to the overall protection by targeting native LT, making it more difficult to evaluate the effect of the adjuvant on the induction of protective immunity separately from the vaccine antigen.

Identifying a suitable wild-type ETEC strain and the optimal dose represents pivotal steps in developing a human challenge model. The strain should be safe to ingest and the doses should be high enough to ensure that most immunologically naïve volunteers develop diarrhea, while not so high as to risk overwhelming an otherwise protective vaccine-induced immunity [16]. In future vaccine challenge trials with ST-based vaccine candidates, volunteers will first receive either the vaccine or placebo and subsequently be experimentally infected with a suitable ST-only ETEC strain. If the ST-based vaccine candidate is efficacious, the vaccinated volunteers should be healthier after being challenged with the ETEC strain than the volunteers who received the placebo vaccination. Until now, two ST-only strains have been tested in volunteer experimental infection studies, including the STp-only 214–4 strain [17] and the STh-only TW11681 strain [18]. Neither of them are optimal because infections with STp-producing strains are not usually associated with moderate-to-severe diarrhea among LMIC children [10, 11], and experimental infection with the STh strain only gave mild diarrhea and a low diarrhea attack risk in volunteers [18].

The main goal of the present study was to evaluate whether the epidemiologically relevant STh-only ETEC strain TW10722 would be safe and useful for testing ST-based ETEC vaccine candidates in human challenge trials. We here assess safety, identify the optimal dose, and report on clinical outcomes and immune responses following experimental infection with strain TW10722.

## Materials and methods

### Volunteers and study setting

We recruited 21 healthy students from the University of Bergen (UiB), Bergen, Norway, who had no history of travel to LMICs during the previous 12 months. The volunteers were recruited on the UiB campus through oral and written information about the project, and those interested were individually given in-depth oral and written information. Before obtaining a written informed consent for participation in the study, the volunteers were given a written questionnaire to make sure they understood the rationale and requirements of the study, including procedures to be undertaken and the potential risks. A description of the inclusion and exclusion criteria, as well as the enrollment process, has been described in detail earlier [18]. The study was conducted at the Infectious Diseases (ID) ward at the Division for Infectious Diseases at Haukeland University Hospital (HUH) in Bergen between 2014 and 2018, with 9 volunteers recruited between September and November 2014 and 12 between September 2017 and March 2018. We included volunteers in groups of three, and each triplet shared a cohort isolation room for up to 10 days after dose ingestion.

### Strain description

ETEC strain TW10722, with serotype O115:H5, was isolated in Guinea-Bissau in 1997 from a 15-month old child suffering from acute diarrhea [11] and kept frozen at -70°C afterwards. It is sensitive to ciprofloxacin, chloramphenicol, and gentamycin, and expresses STh, but not LT or STp. It also expresses the two ETEC colonization factors coli surface antigen 5 (CS5) and 6 (CS6), and is EtpA negative [19]. Strain TW10722 has been shown to produce ST in *in-vitro* assays (Jacob P. Bitoun, personal communication). The strain’s genome has been sequenced (GenBank BioProject no.: PRJNA190209), and results from phylogenetic analyses indicate that it is a good representative of an ETEC family that contributes substantially to childhood diarrhea in LMICs (ETEC5 [20]; L5 [21]).

### Dose preparation

A working cell bank of TW10722 was prepared by the Inoculum Preparation Laboratory at the Center for Vaccine Development and Global Health, University of Maryland School of Medicine, Baltimore, MD, and shipped on dry ice to the study site in Bergen, Norway. The doses given to the volunteers were prepared from these working cell banks similarly to what has been described earlier [18]. Briefly, cells from a vial of frozen working cell bank culture were streaked onto three agar plates that had been prepared with BD Difco Select APS Luria-Bertani animal product-free broth (APF-LB; Becton Dickinson, Franklin Lakes, NJ). Following overnight incubation at 37°C, two colonies from each of the three plates were picked and suspended together in 0.7 mL sterile phosphate buffered saline (PBS). We spread 100 μL of this suspension onto six (for preparing 1×10^10^ colony forming units (CFU) doses) or three (for preparing smaller doses) approximately 10 mm thick APF-LB agar plates prepared in 90 mm petri dishes and incubated them at 37°C for approximately 19 hours, until 2 hours before the volunteers were to ingest the doses. Cells were harvested by scraping from three plates and pooled in a 15 mL Falcon tube containing 10 mL cold PBS. For 1×10^10^ CFU doses, we prepared two tubes (from 6 plates). After resuspending the cells by vortexing, we centrifuged the tubes at 2,000 × g for 5 min at 4°C, poured off the supernatant, and added 10 mL cold PBS. This washing procedure was repeated twice before cell concentration of the resulting stock solution was estimated by measuring the optical cell density at 600 nm (OD600) of diluted stock solution in 10 mm path length cuvettes. We aimed for OD600 measurements between 0.2 and 0.9 and used a conversion factor of 0.9×10^9^ CFU/mL/OD600 to obtain the estimated cell concentration. The stock solution was subsequently diluted in PBS so that each dose was contained in 2 mL PBS suspension. The actual dose given was checked by preparing 10-fold dilution series of the suspension and plating three appropriate dilutions onto LB agar plates in triplicates. This was done both before the doses left the laboratory and after the remaining solutions were returned from dose ingestion at the clinical ward. The 18 plates were incubated overnight at 37°C and the dose confirmed by colony counting. In this study, we prepared separate doses for each group of 3 volunteers. The actual doses given to the volunteers were 1.01×10^6^ CFU (for the 1×10^6^ CFU dose group), 1.00×10^7^ CFU (for the 1×10^7^ CFU dose group), 0.97×10^8^ CFU (for the 1×10^8^ CFU dose group), 1.46×10^9^ CFU (for the 1×10^9^ CFU dose group), and 0.77×10^10^, 0.86×10^10^, and 0.87×10^10^ CFU (for the three 1×10^10^ CFU dose groups).

### Experimental infection and follow up

The volunteers ingested TW10722 in groups of three at a time, starting at a low dose of 1×10^6^ CFU for the first group, as TW10722 had previously not been tested in humans. The dose would, if none or only one volunteer developed diarrhea, be increased 10-fold for the next group, provided the senior study physician (KH) considered it safe to do so. Before dose ingestion, the volunteers fasted from midnight until the dose was given at around 11:00 am the following day. The volunteers first drank 120 mL 1.33% bicarbonate buffer while 30 ml 1.33% bicarbonate buffer was added to the 2 ml dose. After one minute, the volunteers ingested this suspension, and they could eat and drink normally 1 hour afterwards. The infection was cleared by administering 500 mg ciprofloxacin two times daily for three days starting 5 days after the dose was ingested. Antibiotic treatment was started earlier if a volunteer experienced severe diarrhea or moderate diarrhea lasting for ≥24 hours, if a volunteer had mild diarrhea accompanied by two or more non-diarrheal symptoms (fever, vomiting, abdominal pain or cramping, headache, myalgias or nausea) for two days, or if it was considered necessary for other reasons by the senior study physician. Stool specimens (rectal swabs, if stool specimens were unavailable) were collected and screened for the presence of ETEC at least once each day. The specimens were plated on lactose agar and incubated overnight in ambient air at 35°C. A representative selection of *E*. *coli*-like colonies from the plate were pooled and the presence of the challenge strain was determined by detecting the ST gene using real-time PCR as described earlier [22]. The volunteers were kept at the ID ward under the hospital’s enteric precaution guidelines until antibiotic treatment had started and three consecutive stool specimens were negative for ETEC. For all volunteers, we collected blood by venipuncture immediately before dose ingestion as well as 10 and 28 days after. For the 9 volunteers recruited first to the study, we obtained long-term follow-up samples at 2 years after dose ingestion, while for the 12 volunteers recruited last we obtained samples after 6 months and 1 year.

### Clinical assessment

We recorded the volunteers’ vital signs and assessed their physical health and wellbeing immediately before dose ingestion and at least three times daily thereafter. We also performed a daily review of the symptoms noted by each volunteer on a self-report form. Here, the volunteers registered any nausea, abdominal pain or cramping, flatulence, bloating, vomiting, constipation, decreased appetite, headache, malaise, fever, chills, myalgias, and lightheadedness, and graded them as being mild (relieved by using relevant treatment and/or resulting in no disruption of normal daily activities), moderate (only partially relieved by relevant treatment and resulting in some disruption of daily activities), or severe (not relieved by relevant treatment and resulting in disruption of daily activity) [23]. Volunteers who had an axillary temperature reading of ≥38.0°C (measured by using a Bosotherm Basic thermometer [Bosch + Sohn GmbH und Co., Jungingen, Germany]) were classified as having fever. All stools produced by the volunteers were collected and weighed in single-use plastic toilet receptables. As previously described, the stools were graded based on whether it was firm and formed (Grade 1), soft and formed (Grade 2), viscous opaque liquid or semiliquid (Grade 3), opaque liquid (Grade 4) or clear or translucent liquid (Grade 5). An episode of diarrhea was defined as the passing of 1 loose/liquid stool (Grade ≥3) totalling ≥300 g, or ≥2 loose/liquid stools totalling ≥200 g during any 48-hour period within 120 hours after the volunteer had ingested the dose. The severity of each diarrheal episode was further graded as being mild (1–3 loose stools totalling 200–400 g/24 h), moderate (4–5 loose stools totalling 401–800 g/24 h) or severe (≥6 loose stools totalling ≥801 g/24 h) [23]. Based on a combined scoring of symptoms, signs and diarrheal severity, we estimated the disease severity score of each episode, ranging from 0 (least severe) to 8 (most severe) [24].

### Immunoassay antigen preparation

We used PCR to amplify the genes that encode the structural subunits of two ETEC colonization factors CS5 (*csfA*) and CS6 (*cssA*) produced by TW10722, as well as the gene for its *E*. *coli* mucinase YghJ *(yghJ)*. YghJ is a 170 kDa protein that pathogenic *E*. *coli* commonly secrete to break down the protective mucus barrier on the small intestinal epithelium [25]. CS5 and CS6 help anchor ETEC to the intestinal epithelial cells. The structural part of CS5 is made up of multiple repeats of the major subunit CsfA (19 kDa), and the structural part of CS6 is made up of two subunits of similar structure, CssA (15 kDa) and CssB (16 kDa) [26]. The UniProtKB reference accession numbers for the proteins used in immunological assays in the present study are P33781 (YghJ), P0CK95 (CsfA) and P53508 (CssA). The relevant PCR fragments were ligated into pET-30 (for CsfA and YghJ) and pET-32 (for CssA) expression vectors and transformed into ClearColi BL21(DE3) (Lucigen Corp., Middleton, WI), which produces genetically altered LPS that do not trigger unwanted endotoxic responses in T cell assays. To express the proteins, cells were cultured for 50 hours at room temperature in the presence of isopropyl β-D-1-thiogalactopyranoside. Cleared lysates and inclusion bodies were generated by enzymatic lysis (lysozyme) and centrifugation. CsfA and CssA were purified from inclusion bodies, while YghJ was purified from cleared lysates. Proteins in inclusion bodies were denatured by using urea and subsequently renatured by diluting the urea concentration. All proteins were subsequently purified by using HisPur Ni-NTA Resin (Thermo Fisher Scientific, Waltham, MA) according to manufacturer’s instruction. The eluted proteins were dialyzed over night against PBS across a 10 kDa molecular weight cut-off membrane, the protein concentrations were determined by using the Pierce Micro BCA Protein Assay Kit (Thermo Fisher Scientific), and we assessed the quality of the purified proteins by using SDS-PAGE analyses. In those analyses, we confirmed the presence of proteins that had the predicted sizes of CsfA, CssA, and YghJ, and that, by analyzing band signal intensities, these proteins represented >90% of the total peptide content in the solution. We found little or no sign of protein degradation. The proteins were stored in low protein binding tubes at -20°C until use. In the text, we use “CS5” and “CS6A” to refer to CsfA and CssA, respectively.

The CS6 fusion protein (CssAdsB-CssBdsA) consists of the CssA subunit complemented by the donor strand of the CssB subunit (dsB) and the CssB subunit complemented by the donor strand of the CssA subunit (dsA) (S1 Fig). We consider the CS6 fusion protein to be more representative of the CS6 antigens produced by the strain than the CS6A preparation, but we have used CS6A in the T cell assays because the fusion protein was not available during the initial part of the study. The construction of the pET-CssAdsB-CssBdsA plasmid expressing this afimbrial ETEC surface antigen has been previously described [27]. pET-CssAdsB-CssBdsA was transformed into Endotoxin-Free ClearColi BL21 (DE3) cells (Invitrogen, USA). *E*. *coli* transformants were cultivated in Luria-Bertani (LB)-medium containing 100 μg/ml of ampicillin at 37°C. Cells were grown to an OD of 1.4 at 600 nm and induced with 1 mM isopropyl *β*-D-1-thiogalactopyranoside (IPTG) as a final concentration for protein expression. The culture was further grown for 4 hours and expressed proteins were extracted by osmotic shock [28]. The periplasmic fraction (60 ml) was dialyzed twice against 1 liter of 20 mM Tris-HCl, pH 7.8 buffer before purification. The protein was purified by an anion exchange chromatography using a Source Q column in 20 mM Tris-HCl buffer, pH 7.8 at 4°C. A 0–300 mM gradient of NaCl was used to elute the protein. Fractions containing the target protein were pooled and dialyzed overnight in 20 mM Tris-HCl, pH 7.5 buffer. Further purification was performed by another anion exchange column in 20 mM Tris-HCl, pH 7.5 using a Mono-Q column (GE Healthcare) with a 0–200 mM gradient of NaCl at 4°C. Fractions containing CssAdsB-CssBdsA were pooled and dialyzed in PBS-buffer for later use. In the text, we use “CS6AB” to refer to the CssAdsB-CssBdsA fusion protein.

### T cell assay

To investigate antigen-specific CD4+ T cell responses to the experimental infection, we incubated 500 μL sodium-heparinized whole blood with 10 μg purified CS5, CS6A or YghJ for 2 days, and then counted CD25- and CD134 (OX40)-expressing CD4+ T cells using flow cytometry [29, 30]. The T cell assay methodology has previously been described in detail [18]. Briefly, for all volunteers and blood sampling time points, we cultured cells in sodium-heparinized whole blood in X-VIVO 15 Serum-free Hematopoietic Cell Medium with Gentamicin and Phenol Red (Lonza Ltd, Basel, Switzerland) containing 10 μg purified protein/mL. Staphylococcal Enterotoxin B (SEB; 0.1 μg/mL) (Sigma-Aldrich, St. Louis, Missouri) was used as a positive control, and cells cultured in medium only were used as a negative control. The reagents (minus the blood cells) were mixed and frozen at -80°C in 500 μl aliquots in 24-well cell culture plates (Corning Incorporated, Corning, NY) and thawed before use. Specimens from the same volunteer were analysed by using reagents from the same frozen batch, except that new mixes containing new preparations of antigens were made for analysing the 2 year follow-up samples. For each analyses, we added 500 μl blood to each reagent mix and, after incubating for 42–48 hours at 37°C and 5% CO_2_, we added a hypotonic buffer to lyse erythrocytes, and subsequently stained the remaining cells by adding fluorescently-labelled antibodies targeting CD3, CD4, CD8, CD14, CD25 and CD134 (S1 Table), as well as 7-AAD Cell Viability Solution (BioLegend, San Diego, CA). Live singlet CD4+ T lymphocytes were identified by using an LSR Fortessa flow cytometer (BD Biosciences, San Jose, CA). We collected a minimum of 50,000 events in the lymphocyte gate (S2 Fig). We used FlowJo, version 10.5.3 (FlowJo LLC, Ashland, OR) to estimate the percentage of cells co-expressing CD25 and CD134 as a measure of activated antigen-specific CD4+ T cells [30] (S2 Fig).

### Antibody assay

To explore the serum antibody responses to the experimental infection, we performed a multiplex bead-based flow cytometric immunoassay to measure antibody levels against CS5, CS6AB, and YghJ. The methodology has previously been described in detail [18]. Briefly, CS5, CS6AB, YghJ, and the negative control glutathione S-transferase (GST) were covalently coupled to 5 μm Ø Cyto-Plex carboxylated beads (Thermo Fisher Scientific) of different fluorescence intensities. The beads were subsequently incubated with 1:50-diluted serum before incubation with fluorescently-labeled secondary antibodies that recognize human IgA (Alexa Fluor 488-AffiniPure Goat Anti-Human Serum IgA [Jackson ImmunoResearch] and human IgG (Goat anti-human IgG (H+L) Cross-Adsorbed Secondary Antibody, Alexa Fluor 555 [Invitrogen, Waltham, Massachusetts]). Serum collected from all 21 volunteers on day 0, 10 and 28, as well 6 months (n = 12) and 2 years (n = 9) following dose-ingestion were included in the analyses (1-year samples were not available at the time we performed this assay). Fluorescence levels of the labeled beads were measured on an LSR Fortessa flow cytometer. As a measure of protein-specific serum antibody levels, we used FlowJo to calculate the median fluorescence intensity (MFI) of the beads for each protein and subtracted the corresponding MFI value of the GST-labeled negative control beads.

### Statistical analyses

We tested for differences in antigen-specific CD4+ T cell and antibody levels between blood specimens collected at different time points using the Wilcoxon matched-pairs signed rank test in GraphPad Prism (GraphPad Software Inc., La Jolla, CA). To estimate the association between CD4+ T cell or antibody levels, dose, and the presence of diarrhea, we performed multiple linear regression analyses using SPSS (IBM SPSS Statistics for Windows Version 25.0, Chicago, Illinois). In these regression models, we included the target dose (CFU) and experiencing a diarrheal episode (yes/no) as independent variables, and the fold-change from day 0 to day 10 or 28 in antibody or CD4+ T cell levels as the dependent variable. Fold changes represent the ratio between the measured (i.e. day 10 and 28) and the baseline (i.e. day 0) values. We used linear regression to examine the association between anti-CS5, -CS6AB, and -YghJ antibody levels and the corresponding antigen-specific CD4+ T cell responses. For each tested antigen, we included the log10 transformed absolute difference in CD4+ T cell levels from day 0 to day 10 as the independent variable, and the log10 transformed absolute difference in serum IgG or IgA peak levels (from day 0 to day 28 [CS5] or from day 0 to day 10 [for CS6AB and YghJ]), was included as the dependent variable. Absolute differences were calculated by subtracting the baseline (i.e. day 0) from the measured (i.e. day 10 and 28) values. The significance threshold was set at p <0.05.

### Ethics statement

All volunteers gave an informed written consent before being included in the study. The study is approved by the Regional Committee for Medical and Health Research Ethics, Health Region West (Project ID: 2014–826), and registered in ClinicalTrials.gov (Project ID: NCT02870751). The study was assessed by an independent safety monitor before, during and after enrollment of volunteers to ensure adherence to Good Clinical Practice (GCP) standards.

## Results

### Volunteer characteristics

**Fig 1:**
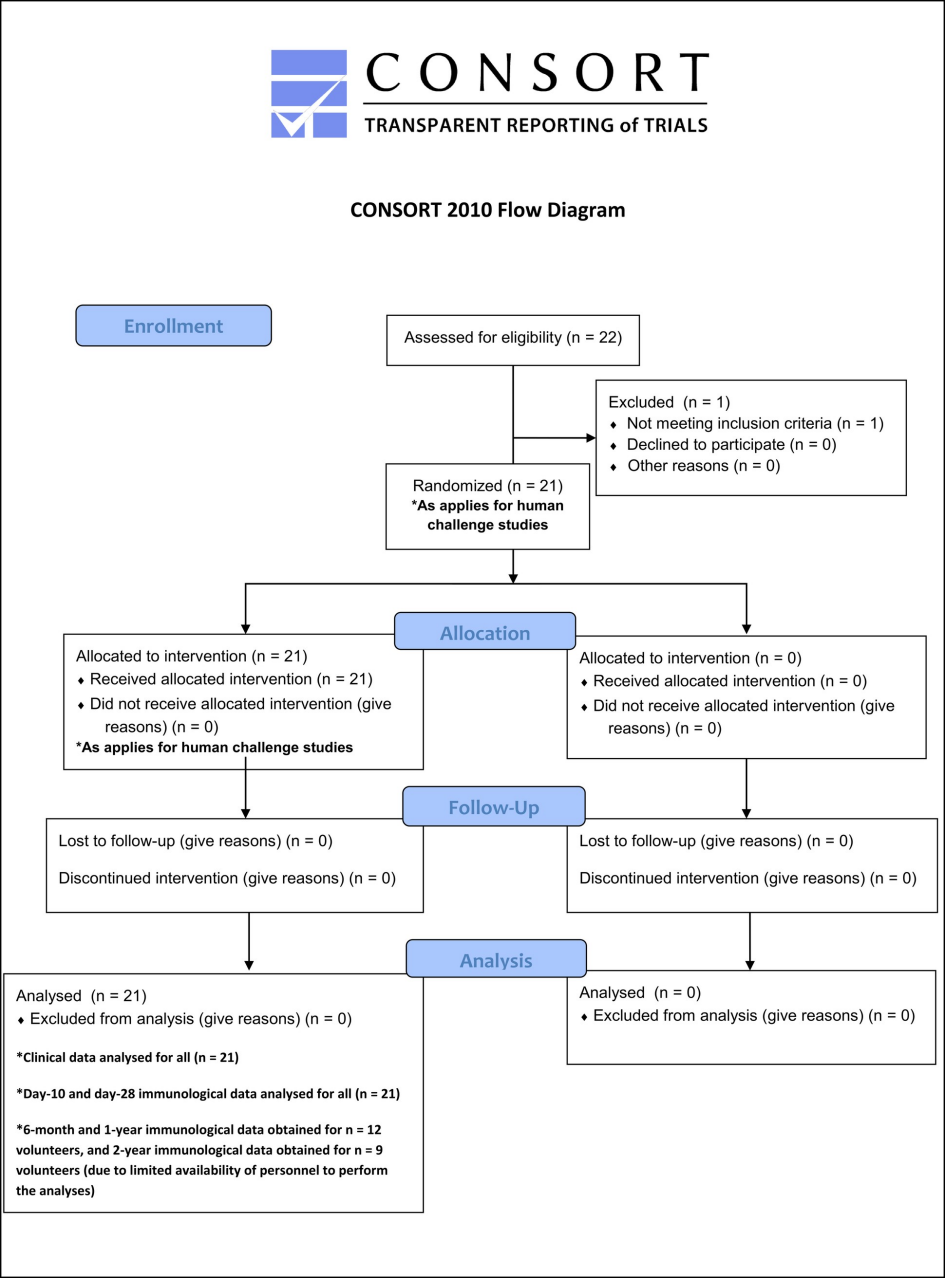
Flow diagram. CONSORT flow diagram as applies for human challenge studies. In total 22 adult volunteers were assessed for eligibility, and 1 volunteer did not meet the inclusion criteria, resulting in a total of 21 volunteers allocated to experimental infection with different doses of ETEC: 1×10^6^ (n = 3), 1×10^7^ (n = 3), 1×10^8^ (n = 3), 1×10^9^ (n = 3) and 1×10^10^ (n = 9) CFU. Clinical data were analyzed for all volunteers, as well as the immunological data on day 10 and day 28 after experimental infection. There were none lost to follow-up, however, immunological data from 6 months, 1 year and 2 years after experimental infection were obtained from subgroups of the study population due to limited availability of personnel to perform the analyses.

A total of 21 healthy adult volunteers, of which 18 were women, were enrolled for experimental infection with strain TW10722 (Fig 1). They were 19 to 29 years old and had a body mass index (BMI) varying from 19.3 to 27.4 kg/m^2^. Among the 12 volunteers from which we obtained 1-year samples, 2 had been traveling in ETEC endemic countries since the previous follow-up, while 8 of the remaining 9 volunteers from whom we obtained 2-year samples had traveled to such countries.

### Dose optimization and clinical outcomes

For the 21 volunteers included in this study, we administered the following doses: 1×10^6^ (n = 3), 1×10^7^ (n = 3), 1×10^8^ (n = 3), 1×10^9^ (n = 3) and 1×10^10^ (n = 9) CFU. Up to and including doses of 1×10^9^ CFU, at most one in three volunteers developed diarrhea (Table 1). At 1×10^10^ CFU doses, the volunteers more consistently developed diarrhea, with 2 out of 3 volunteers developing diarrhea in the first enrolled triplet, and a total 7 out of 9 volunteers (78%, 95% confidence interval [CI]: 44% to 95%) falling ill, 6 of whom experienced moderate or severe diarrhea. Among the volunteers who developed diarrhea, each 10-fold increase in dose was associated with 11.6 hours (95% CI: 8.3 to 14.9) shorter incubation period. Three volunteers, all of whom experienced severe diarrhea, received early antibiotic treatment. The volunteers who received doses ranging from 1×10^6^ to 1×10^9^ CFU had a similar age distribution and similar BMI measurements compared to those given 1×10^10^ CFU doses.

**Table 1:** Description of diarrheal episodes among 21 volunteers experimentally infected with ETEC strain TW10722.

Dose (CFU)	No. of volunteers	No. with diarrhea	Attack risk	Median severity	Mean incubation period, hours (range)	Mean 24h maximum stool output, grams (range)	Mean whole episode stool output, grams (range)	Mean episode duration, hours (range)	Mean 24h maximum stool output, count (range)
1 × 10^6^	3	0	0%	NA*	NA*	NA*	NA*	NA*	NA*
1 × 10^7^	3	1	33%	Mild	58	286	286	0	1
1 × 10^8^	3	1	33%	Severe	44	1773	2754	42	11
1 × 10^9^	3	1	33%	Severe	46	543	543	13	7
1 × 10^10^	9	7	78%	Moderate	23 (18–26)	492 (389–711)	496 (389–711)	14 (0–66)	4 (1–8)

* NA: Not applicable.

For all volunteers, nausea, malaise and headache were frequently occurring symptoms, and they were observed more often at higher doses (Table 2). Apart from three episodes of moderate or severe lightheadedness, however, the symptoms were all reported to be mild or moderate, which contributed to relatively low disease severity scores (Table 3). Two volunteers developed mild fever (both 1×10^8^ CFU doses), and two experienced vomiting (both 1×10^10^ CFU). There were no serious adverse events, and none of the volunteers needed administration of oral rehydration salts solution or intravenous fluid.

**Table 2:** Symptoms and signs other than diarrhea in 21 volunteers experimentally infected with ETEC strain TW10722.

Symptom	Dose (CFU)		
	1×10^6^ – 1x10^9^ n = 12	1x10^10^ n = 9	Combined n = 21
Nausea	4 (33)	6 (67)	10 (48)
Abdominal pain	5 (42)	6 (67)	11 (52)
Abdominal cramping	2 (17)	5 (56)	7 (33)
Excessive flatus	4 (33)	7 (78)	11 (52)
Decreased appetite	1 (8)	2 (22)	3 (14)
Bloating	4 (33)	7 (78)	11 (52)
Vomiting	0 (0)	2 (22)	2 (10)
Constipation	0 (0)	1 (11)	1 (5)
Headache	4 (33)	5 (56)	9 (43)
Malaise	3 (33)	6 (67)	10 (48)
Fever	2 (17)	0 (0)	2 (10)
Chills	1 (8)	0 (0)	1 (5)
Generalized myalgias	1 (8)	0 (0)	1 (5)
Lightheadedness	3 (25)	2 (22)	5 (24)
Hypovolemia	0 (0)	0 (0)	0 (0)

The table displays the number of volunteers experiencing each symptom or sign (percentages in parentheses).

**Table 3:** Mean disease severity scores among 21 volunteers experimentally infected with ETEC strain TW10722.

Dose (CFU)	N	Mean sub-score			Mean disease severity score (0–8)
		Objective signs (0–2)	Subjective symptoms (0–2)	Diarrhea score (0–4)	
1 × 10^6^	3	0.0	0.3	0.0	0.3
1 × 10^7^	3	0.0	1.0	0.3	1.3
1 × 10^8^	3	1.3	1.0	1.3	3.7
1 × 10^9^	3	0.0	0.7	0.7	1.3
1 × 10^10^	9	0.2	1.0	1.6	2.8

### CD4+ T cell responses

**Fig 2:**
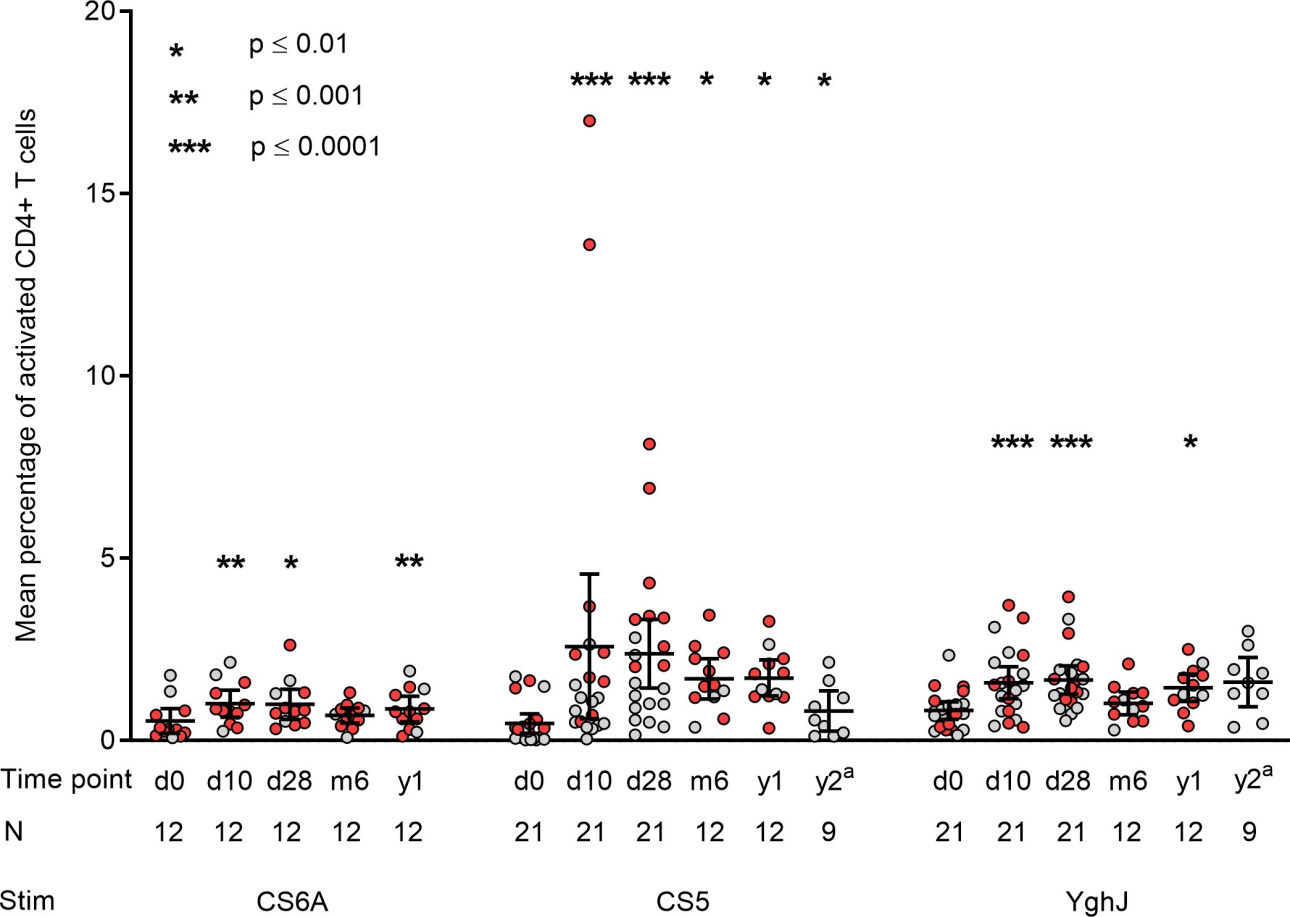
Antigen-specific CD4+ T cell responses after infection with STh-only ETEC strain TW10722. The graph displays the mean percentage of CD4+ T cells that co-express CS25 and CD134 for each sampling timepoint and virulence factor after incubating whole blood with ETEC proteins CS6A, CS5 and YghJ. Grey circles represent volunteers in the 1×10^6^ to 1×10^9^ CFU dose groups, red circles represent volunteers in the 1×10^10^ CFU dose group. Error bars represent the 95% confidence intervals. Due to limited availibility of purified CS6 antigen in the initial parts of the study, CS6-specific CD4+ T cell responses were only measured for a subgroup of the volunteers (n = 12). Also, due to the stepwise inclusion of volunteers to the study, the long-term follow-up timepoints of each volunteer varied according to time of enrollment, with the first subgroup of volunteers (n = 9) having their follow-up at 2 years, while the last subgroup of volunteers (n = 12) had their follow-up at 6 months and 1 year after experimental infection. Abbreviations: d0: day 0, d10: day 10, d28: day 28, m6: 6 months, y1: 1 year, y2: 2 years, N: Number of volunteers, Stim: Antigen used for stimulation. ^a^ Antigen preparation differed from other timepoints.

Following incubation of peripheral blood with CS5, we found a mean 5.5-fold increase in the percentage of activated antigen-specific CD4+ T cells, from 0.47% on day 0 to 2.58% on day 10 (p < 0.0001) (Fig 2). Although the levels were slightly reduced after this, they remained markedly elevated both 28 days (5.1-fold, p < 0.0001) and 6 months after dose ingestion, and even in the 1- and 2-year follow-up samples. In total, there were 20 volunteers (95%) who responded with a ≥ 2.0-fold and 16 volunteers (76%) with a ≥ 4.0-fold increase in the percentage of activated CD4+ T cells after CS5 stimulation.

Following incubation of peripheral blood with CS6A and YghJ we also observed increases in the percentage of activated antigen-specific CD4+ T cells, but the responses were generally weaker and less long-lived than those seen for CS5; a maximum 1.9-fold increase for CS6A (p < 0.001) from day 0 to day 10 and a maximum 2.0-fold increase (p < 0.0001) for YghJ from day 0 to day 28. In total there were 10 volunteers (83%) who responded with a ≥ 2.0-fold increase in the percentage of CS6A-specific CD4+ T cells, and 16 volunteers who responded with the same fold increase in the percentage of YghJ-specific CD4+ T cells. Purified CS6A was not available in the initial part of the study, which is why this antigen was only tested in samples from the 12 last recruited volunteers (Fig 2).

Averaged across all volunteers and sampling timepoints, whole blood incubation with medium only (negative control) activated a mean 0.03% of CD4+ T cells, while incubation with Staphylococcal Enterotoxin B (positive control) activated a mean of 14.5%.

### Serum antibody responses

**Fig 3:**
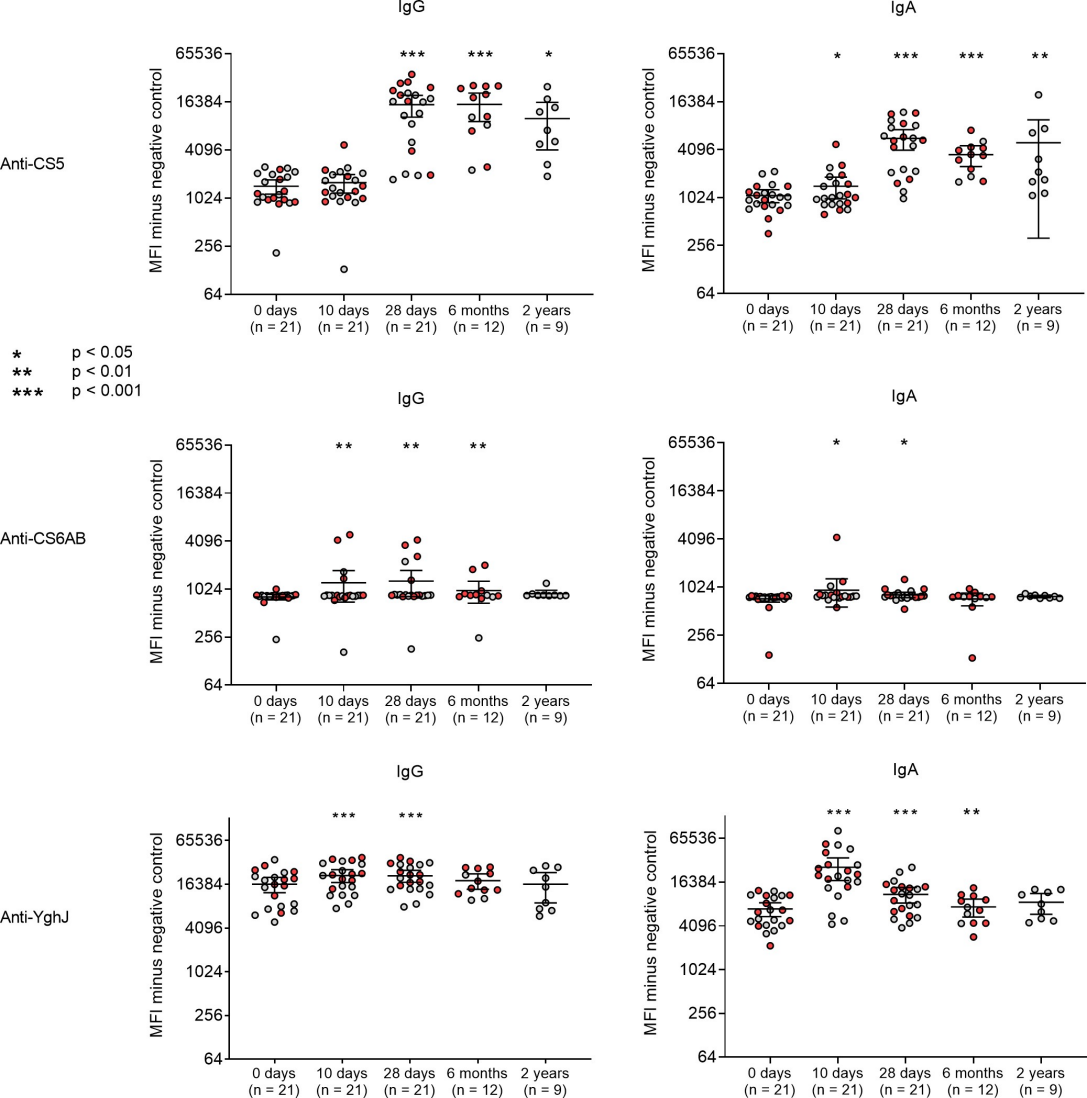
Antigen-specific serum IgG and IgA responses after infection with STh-only ETEC strain TW10722. The graphs display the median fluorescence intensity (MFI) value of CS5-, CS6AB-, and YghJ-specific IgG and IgA at each sampling time point for each volunteer. Grey circles represent volunteers in the 1×10^6^ to 1×10^9^ CFU dose groups, red circles represent volunteers in the 1×10^10^ CFU dose group. Error bars represent the 95% confidence intervals. Number of volunteers indicated in parentheses. Abbreviations: d0: day 0, d10: day 10, d28: day 28, m6: 6 months, y2: 2 years.

Levels of serum antibodies targeting CS5 increased substantially from day 0 to day 28, with a mean 10.5-fold MFI increase in anti-CS5 IgG (p < 0.0001) and a mean 5.2-fold MFI increase in anti-CS5 IgA (p < 0.0001) (Fig 3). Six months after the experimental infection, the antibody levels remained very high for IgG, and somewhat less, yet still significantly so, for IgA. In total, there were 17 volunteers (81%) who developed a ≥ 4.0-fold increase in anti-CS5 IgA levels, and correspondingly 12 volunteers (57%) with the same fold increase in anti-CS5 IgG levels. Elevated antibody levels could be detected even 2 years after dose ingestion, with a mean 6.0-fold increase from baseline in IgG (p = 0.008) and a 4.1-fold increase from baseline in IgA (p = 0.004) compared to the day 0 levels.

Correspondingly, we measured significant increases in serum antibody levels targeting YghJ (Fig 3). These developed faster than the CS5 responses, but were generally lower and more short-lived, and markedly more consistent for IgA levels (17 volunteers [81%] with ≥ 2.0-fold increase) compared to IgG levels (3 volunteers [14%] with ≥ 2.0-fold increase). A modest mean increase of 1.3-fold in anti-YghJ IgG was observed from day 0 to day 10 (p < 0.0001), and the levels were stably elevated to day 28 before dropping to baseline levels 6 months after dose ingestion. An equally rapid, but stronger response was seen for anti-YghJ IgA, with a 3.7-fold increase from day 0 to day 10 (p < 0.0001) before levels gradually declined towards 28 days and 6 months after dose ingestion.

The anti-CS6AB antibody increases were generally weaker than for CS5 and YghJ, and the responses seemed to be more heterogeneous, with some volunteers having peak anti-CS6AB antibody levels on day 10, while others had peak levels on day 28, or appeared to remain largely unresponsive throughout the follow-up period. In total, there were 2 volunteers (10%) who developed a ≥ 2.0-fold increase in anti-CS6AB IgG, and correspondingly 5 volunteers (24%) with the same fold increase in anti-CS6AB IgA levels. Overall, we observed a mean 1.5-fold increase in anti-CS6AB IgG (p = 0.006) and a 1.3-fold increase in anti-CS6AB IgA (p = 0.014) from day 0 to day 10.

### Associations between clinical symptoms and immune responses

Results from multiple linear regression analysis showed that volunteers developing diarrhea (n = 10) tended to have higher increases in antigen-specific antibody and CD4+ T cell levels compared to volunteers who did not develop diarrhea (n = 11). However, only the increase from day 0 to day 28 in anti-CS5 IgA (p = 0.036), as well as the increase from day 0 to day 10 in YghJ-specific CD4+ T cells (p = 0.033) were significantly associated with developing diarrhea. There was no clear association between inoculation dose and CS5-, CS6-, or YghJ-specific antibody or activated CD4+ T cell levels.

### Associations between antibody and CD4+ T cell responses

Linear regression analysis using log10 transformed values showed that increases in CS5- and CS6AB-specific antibody levels were associated with increases in the corresponding antigen-specific CD4+ T cell responses. The association was significant for anti-CS5 IgA (p = 0.023) and anti-CS6AB IgA (p = 0.048), and approaching significance for anti-CS5 IgG (p = 0.051) and anti-CS6AB IgG (p = 0.068). No significant association was found between the humoral and cellular YghJ-specific responses.

## Discussion

With the aim to develop a human challenge model that can be used to estimate the protective efficacy of ST-based vaccine candidates in Phase 2B trials, we set out to identify a suitable epidemiologically relevant ST-only ETEC strain that would be safe to administer to volunteers and induce diarrhea in around 70% of them. We here demonstrate that a dose of 1×10^10^ CFU yielded a suitable overall diarrhea attack risk of 78% (95% CI: 40% to 97%) and a moderate or severe diarrhea attack risk of 67% (95% CI: 30% to 93%). Although experimentally infecting more volunteers with this dose would have improved the precision of the attack risk estimates, we believe they are good approximations of the true attack risks for this model. Nevertheless, their precision will improve as actual vaccine challenge studies generate data.

We also demonstrated that it is safe for healthy human volunteers to ingest ETEC strain TW10722 at 1×10^10^ CFU. The non-diarrheal symptoms associated with this dose were mostly mild or moderate, and none of the volunteers experienced any severe adverse events or needed oral rehydration salts or intravenous solutions. Finally, most volunteers elicited strong specific antibody and CD4+ T cell responses against the TW10722 colonization factors CS5 and CS6, as well as against the *E*. *coli* mucinase YghJ. Our results indicate that a human challenge model based on a 1×10^10^ CFU dose of strain TW10722 will be safe and efficient to use in a trial estimating the protective efficacy of ST-based vaccine candidates for diarrhea among LMIC children and in travelers. The model should also be suitable for evaluating the protective efficacy of vaccine candidates that target CS5 and CS6. In addition, since strain TW10722 does not produce LT, the model may be useful for evaluating the effects of LT-based adjuvants, such as dmLT.

ETEC strain TW11681, which expresses colonization factor antigen I (CFA/I) and CS21, is the only other STh-only ETEC strain that has been used in human volunteer studies [18]. Both strains TW11681 and TW10722 belong to epidemiologically relevant ETEC families that are commonly associated with diarrhea among LMIC children [20, 21]. When given to volunteers in doses between 1×10^6^ and 1×10^8^ CFU, which normally give relatively high attack risks for strains that produce both STh and LT [31], both these strains rarely elicited diarrhea. Within the groups receiving the highest doses (1×10^8^, 1×10^9^ or 1×10^10^ CFU), the clinical presentation typically ranged from no symptoms, to severe diarrhea with additional symptoms such as abdominal cramping, mild fever and/or vomiting. This variation in disease severity, from mild to severe, has earlier been pointed out to be a characteristic feature of challenge strains producing ST, either alone or in combination with LT [31]. Some data also suggest that the number of guanylate cyclase C (GC-C) receptors in human intestinal membranes decreases with increasing age [32], thus being lower in adults than in infants, which can help explain why some adult volunteers develop no or very mild symptoms to ST-only ETEC infection. Another potential explanation for the variation in symptoms we observed is that some volunteers could lack receptors the TW10722 strain needs to properly colonize the small intestine. Although variation in these receptors has not yet been seen in humans, it is recognized as an important source of variation in symptoms among ETEC-infected piglets [33, 34]. Finally, the early administration of antibiotic treatment to three volunteers with severe diarrhea in our current study may also have contributed to lowering overall disease severity scores by reducing the total diarrheal stool output.

Following the volunteers’ ingestion of TW10722, we measured immune responses targeting both CS5 and CS6, confirming that both these colonization factors were expressed by the bacteria during the infection. Our study design also allowed a long follow-up of immune responses, and to our knowledge we are the first to describe antigen-specific CD4+ T cell responses as late as 2 years after dose ingestion, although it must be noted that the 2-year measurements were based on cell culturing with a separate stock of purified protein, making interpretation somewhat difficult. CS5 stimulation resulted in strong antigen-specific activation of CD4+ T cells in peripheral blood, which demonstrated the strong antigenic properties of this fimbrial colonization factor. We also observed a prominent increase in serum IgG and IgA targeting CS5, and the response showed remarkable longevity, with some individuals still having elevated antibody levels 2 years after dose ingestion. However, close to all volunteers in this subgroup had been traveling in ETEC endemic countries within this 2-year period, so we cannot rule out the possibility that some of the long-term elevated antibody responses were boosted following more recent enteric infections. The *E*. *coli* mucinase YghJ also elicited strong immune responses, with the anti-YghJ IgA antibody response appearing to be more prominent than the IgG response, similar to what we observed in the sera from the volunteers experimentally infected with the TW11681 strain [18]. However, being highly conserved across different *E*. *coli* strains [25], it is likely that many of the volunteers have been pre-exposed to YghJ, and that the rapid increase in anti-YghJ IgA and IgG represents a recall response. Interestingly, multiple regression analysis showed that development of diarrhea, but not the TW10722 dose size, was significantly associated with increased immune responses. However, the number of volunteers (n = 21) is too small to draw any definitive conclusions.

CS6 is characteristically expressed by ST-positive strains (with or without LT), and it is usually present alone or with CS4 or CS5 [35]. In contrast to most other ETEC colonization factors, CS6 has a non-fimbrial morphology, and its exact function is not yet fully understood, although CS6 has been shown to mediate bacterial adherence to enterocytes [27, 36]. Here, we have provided the first report on CS6-specific immune responses following experimental infection with an STh-only ETEC strain. We found that the mean proportion of CS6-specific CD4+ T cells was significantly increased both 10 and 28 days after ingestion of TW10722, while the increases in anti-CS6 serum antibody levels were generally small and more variable. This is in agreement with results from safety and immunogenicity trials of the oral inactivated ETVAX vaccine candidate showing that only a few Swedish vaccinees (3–19%) developed a ≥ 2-fold increase in plasma anti-CS6 antibody levels after vaccination [37]. Similar low frequencies of CS6 seroconversion have also been observed in adult volunteers after ingesting the oral live-attenuated vaccine candidate ACE527 [38] and the LT-ST-CS6-expressing challenge strain B7A [39]. The absence of immunological priming in ETEC-naïve subjects has been suggested as an explanation to these weak responses [6]. This may also help explain the variable anti-CS6 serum antibody response observed in our Norwegian, presumably relatively ETEC-naïve, volunteers, with only 5 (24%) and 2 (10%) of them developing a ≥ 2-fold increase in serum anti-CS6 IgG and IgA, respectively.

### Conclusions

We here present a safe STh-only ETEC human challenge model based on the epidemiologically relevant strain TW10722 expressing the colonization factors CS5 and CS6. The strain is safe to administer to healthy volunteers, and yielded an attack risk for moderate or severe diarrhea of 67% and an overall diarrheal attack risk of 78% when given in doses of 1×10^10^ CFU. These estimates are based on results from experimental infection of 9 volunteers, and they will improve when the model is used in vaccine challenge studies. The challenge model strain also induced strong antibody responses in serum as well as CD4+ T cell responses in peripheral blood targeting both CS5, CS6 and YghJ, some of which showed remarkable longevity with significantly increased levels 1 year after dose ingestion. In conclusion, strain TW10722 would be suitable for use in Phase 2B vaccine challenge trials for evaluating the efficacy of ST-based vaccines for ETEC diarrhea.

## Supporting information

S1 Checklist STROBE checklist. (DOC)

S1 Table List of antibodies used in T cell assay. (DOCX)

S1 Fig Protein sequence of the CssAdsB-CssBdsA construct. Signal peptide and donor strand sequences are shown in blue and red, respectively. The linker sequence is shown in lowercase.
(DOCX)

S2 Fig Gating strategy used to identify activated antigen-specific CD4+ T cells. Forward and side scatter was used to identify singlet lymphocytes, and monocytes and dead cells were excluded by using the CD14 and 7-AAD signals. CD4+ T cells were identified by the expression of CD3 and CD4, and out of these we determined the percentage of CD25-CD134 doubly positive cells as a measure of activated antigen-specific CD4+ T cells. FMO controls were used to guide the positioning of the CD25+ CD134+ gate. Abbreviations: FMO: Fluorescence Minus One, SEB: Staphylococcal Enterotoxin B.
(PNG)

S1 Data Clinical responses. (XLSX)

S2 Data CD4 T cell responses. (XLSX)

S3 Data Serum responses. (XLSX)

S1 Protocol Trial protocol for experimental infection study describing background, study objectives, material and methods, project management, organization and cooperation, as well as relevant amendments to the protocol. (PDF)
