# Supplementary material for: A new human challenge model for testing heat-stable toxin-based vaccine candidates for enterotoxigenic Escherichia coli diarrhea – dose optimization, clinical outcomes, and CD4+ T cell responses
Source: PLoS Negl Trop Dis. 2019 Oct 30;13(10):e0007823. doi: 10.1371/journal.pntd.0007823 (PMC6844497; doi:10.1371/journal.pntd.0007823)
Supplement: S1 Table — (DOCX) [file pntd.0007823.s002.docx]

**S1 Table. List of fluorescent markers used in T cell assay**

| **Antibody** | **Clone** | **Fluorochrome** | **Supplier** | **Catalog#** |
| --- | --- | --- | --- | --- |
| CD3 | HIT3a | AF700 | BioLegend | 300324 |
| CD4 | OKT4 | BV510 | BioLegend | 317444 |
| CD8a | HIT8a | FITC | BioLegend | 300906 |
| CD25 | M-A251 | APC | BD Biosciences | 555434 |
| CD134 | ACT35 | PE | BD Biosciences | 555838 |
| CD14 | 61D3 | Pe-Cy5 | Thermo Fisher Scientific | 15-0149-42 |
| 7-AAD | - | - | BD Biosciences | 555816 |
