# Supplementary material for: A new human challenge model for testing heat-stable toxin-based vaccine candidates for enterotoxigenic Escherichia coli diarrhea – dose optimization, clinical outcomes, and CD4+ T cell responses
Source: PLoS Negl Trop Dis. 2019 Oct 30;13(10):e0007823. doi: 10.1371/journal.pntd.0007823 (PMC6844497; doi:10.1371/journal.pntd.0007823)
Supplement: S1 Fig — Signal peptide and donor strand sequences are shown in blue and red, respectively. The linker sequence is shown in lowercase. (DOCX) [file pntd.0007823.s003.docx]

**S1 Figure**

MKKTIGLILILASFGSHARTEPVSTTISKSFFAPEPQIQPSFGKNVGKEGGLLFSVSLTVPENVSQVTVYPVYDEDYGLGRLVNTADDSQSIIYQIVDDKGRKMLKDHGAEVTPNQQITFRALNYTSGDKEIPPGIYNDQVMVGYYVNdnkqGNWQYKSLDVNVNIEQNFIPDIDSAVRIIPVNYDSDPKLNSQLYTVEMTIPAGVSAVKIVPTDSLTSSGQQIGKLVNVNNPDQNMNYYIRKDSGAGKFMAGQKGSFSVKENTSYTFSAIYTGGEYPNSGYSSGTYAGHLTVSFYSNdnkqRTEIATKNFPVSTTIS
