# Supplementary material for: A new human challenge model for testing heat-stable toxin-based vaccine candidates for enterotoxigenic Escherichia coli diarrhea – dose optimization, clinical outcomes, and CD4+ T cell responses
Source: PLoS Negl Trop Dis. 2019 Oct 30;13(10):e0007823. doi: 10.1371/journal.pntd.0007823 (PMC6844497; doi:10.1371/journal.pntd.0007823)
Supplement: S1 Protocol — (PDF) [file pntd.0007823.s008.pdf]

EXPERIMENTAL INFECTION OF VOLUNTEERS WITH HEAT-STABLE TOXIN (ST)-ONLY PRODUCING ENTEROTOXIGENIC *ESCHERICHIA COLI* (ETEC): CONTRIBUTING TO THE DEVELOPMENT OF VACCINES AGAINST CHILDHOOD DIARRHEA IN LOW- AND MIDDLE-INCOME COUNTRIES.

In 2013, we completed a study at Haukeland University Hospital where 30 volunteers were experimentally infected with a wild-type ETEC strain TW10598 which produces the thermo-labile toxin (LT) as well as ST. The goal of the study was to develop an ETEC challenge model using a strain from one of the ETEC families that contribute the most to childhood diarrhea in low and middle income countries (LMIC), and to generate biological material that could be used to study immune responses and to identify potential new vaccine antigens. The study was approved by the Regional Committee for Medical and Health Research Ethics, Health Region West (REC-West, case number 2010/728-14). We consider the project a success. The strain was well tolerated by the volunteers, giving few side-effects (manuscript submitted to BMC Infectious Diseases).

As part of the newly funded GLOBVAC project "Development of a heat-stable toxoid-based (ST) vaccine for enterotoxigenic *Escherichia coli* diarrhea (ETECvac)", we now aim to develop a challenge model that can be used for testing the efficacy of ST-based vaccines. Such models do not currently exist, but they are needed for undertaking Phase IIB clinical trials (i.e. challenge studies) of vaccine prototypes that our research consortium is currently developing. For the purpose of developing this challenge model, we wish to undertake a new experimental infection study similar to the one recently completed, but by using a new wild-type ETEC strain (TW10722) with the O115 antigen and that produces ST, but does not produce LT. In addition to evaluating the ETEC-specific humoral immune responses to the infection, like we did in our recently completed experimental infection study, we will now also evaluate cellular immune responses Antibodies from the volunteers will further be used to screen for other ETEC antigens that have induced an immune response during the infection in order to identify new antigens that may be relevant for vaccine development. To quantitate a protective effect of an ST-based vaccine in Phase IIB trials, we will develop and test new methods to quantitate TW10722 and the amount of ST present in the stools of the volunteers. Finally, we wish to look for changes in the intestinal microbiota between before and during an ETEC infection. All studies will be conducted according to the latest version of the Declaration of Helsinki.

Background

Enteric disease continues to be among the most important killers of children in LMIC. Moreover, diarrhea predisposes to malnutrition, which in turn increases susceptibility to other infections, such as pneumonia. Recent data suggest that diarrhea may also contribute to delay

in cognitive development. Over the last 20 years, case management with oral rehydration therapy and continued feeding has substantially decreased diarrhea-related deaths. However, the ~1 million deaths occurring annually still make diarrhea the second leading cause of child death (Walker *et al*., 2007). A principal impediment to diarrheal disease control is the large number of enteropathogens. Certain diarrheal pathogens, including rotavirus, enterotoxigenic *E. coli* (ETEC), *Shigella* flexnerii and *Cryptosporidium parvum* are the most important causes of severe diarrheal disease and death (Kotloff *et al*., 2013). Effective vaccines against ETEC, *Shigella* and *Cryptosporidium* are yet to be developed.

ETEC causes diarrhea by the release of the heat-stable toxin (ST) and/or the heat-labile toxin (LT), and they express colonization factors (CFs) which promote their attachment to the small intestinal epithelium (Walker *et al.*, 2007). Vaccine development during the last decade has mainly targeted the major CFs as well as the immunogenic LT, but they have so far failed to confer adequate protection (Walker *et al*., 2007). ETEC strains producing only ST or both ST and LT contribute more to the disease burden in LMIC children than do those that express only LT (Levine *et al*., 1993; Valentiner-Branth *et al*., 2003; Kotloff *et al*., 2013). Vaccines that induce an effective anti-ST response have not yet been successfully developed because ST is in itself non-immunogenic, and it has been difficult to render it non-toxic and at the same time immunogenic without changing its most important antigenic properties. In addition, ST has some antigenic epitopes that are similar to the human ligands guanylin and uroguanylin (Taxt *et al.* 2014), raising concerns that ST-based vaccines may also induce an immune response against these endogenous ligands. However, progress has now been made in overcoming these challenges (Taxt *et al.* 2014), and we expect that our research team can contribute to the development new ST-based vaccine prototypes, and we hope that these candidates will be ready for testing within the next few years. For this reason, there is now an urgent need to develop a human challenge model employing an ST-only ETEC strain that can be used for testing the efficacy of ST-based vaccine prototypes in coming Phase IIB trials.

Study objectives

Overall objective

Develop a challenge model for testing the efficacy of new ST-based vaccines

Specific objectives

1. Assess the safety and identify the optimal dose for infection with strain TW10722
2. Develop new methods for quantitating strain TW10722 and ST in stools

Additional objectives

3. Characterize human immune responses induced by strain TW10722

4. Screen for ETEC antigens that induce immune responses during experimental infection.
5. Characterize changes in microbiota as a result of ETEC infection

Description of work

1. Assess the safety and identify the optimal dose for infection with strain TW10722

By experimentally infecting presumably immunologically naïve volunteers with wild-type ST-only ETEC strain TW10722, we will assess the safety of ingesting the strain and identify the dose that gives a 70% attack risk of diarrhea, which is suitable for use in Phase IIB trials. We will perform this dose-escalation experimental infection study similar to our previous study, starting with a low (1×10^6 colony-forming units [CFUs]) dosage for the first group of volunteers before gradually increasing the dosage for subsequent groups of volunteers. We expect that the optimal dose will be between 1×10^8 and 1×10^9 CFU, based on our and others' experiences with such studies. The maximum dosage we will attempt is 1×10^{10} CFU. Up to 30 volunteers will be recruited to the study. Informed consent will be collected from volunteers by researches to which the volunteers do not have any relation of dependence. Joining the study is voluntary and the volunteer will be free to withdraw at any time.

The chosen study-strain, TW10722, was isolated from a 15-month old child suffering from diarrhea in Guinea-Bissau in 1997 (Steinsland *et al*., 2002) and is well characterized. It has serotype O115:H5, produces the human variant of ST (STh) as well as the ETEC colonization factors Coli surface antigen 5 (CS5) and CS6, it is susceptible to most relevant antibiotics. We sequenced its entire genome in 2011 (GenBank BioProject no.: PRJNA190209). The strain is an excellent representative of an ETEC family (ETEC5) that contributes substantially to childhood diarrhea in LMIC (Steinsland *et al*., 2010).

A working cell bank of strain TW10722 will be produced by the Inoculum Preparation Laboratory of the Center for Vaccine Development at the University of Maryland School of Medicine and shipped to Bergen on dry ice, as was done for TW105098 for our previous experimental infection study. Doses made from these culture stocks will be prepared fresh for each group of volunteers in the same manner that was performed in our previous study.

All volunteers will receive a detailed explanation of the study protocol, and on screening day they will fill out a questionnaire to ensure they have understood the information given. The screening will be performed in the outpatient clinic a few days prior to infection, where personal data and medical history will be recorded and a physical examination will be performed. The examination will include total blood cell count, measurement of serum concentrations of electrolytes, creatinine, ALT, and glucose, as well as testing for antibodies to hepatitis B and

C, and a combined antibody/antigen assay for HIV infection. Women will undergo a urine-based pregnancy test, and electrocardiograms will be taken. Stool specimens will be checked for occult blood and screened against an array of enteropathogens. The inclusion critera will be age between 18 and 40, not having travelled to low- or middle-income countries for the previous 12 months (if this turns out to be too restrictive we may change this to 9 months), written informed consent, completed general health screening, stool culture free for enteropathogens, normal base-line blood tests, being able to be in isolation for up to 10 days, and effective contraception in the women. The exclusion criteria will be fever (≥38°C) during the last 48 hours before study initiation, allergy to ciprofloxacin or beta-lactam antibiotics, participation in other clinical trials during the last 3 months, use of immunosuppressive agent, pregnancy, breastfeeding, positive fecal occult blood test, or a history of any chronic gastrointestinal conditions.

The volunteers will be admitted to the Unit for Infectious Diseases, Department of Medicine, Haukeland University Hospital, Bergen, in cohorts of 2-4 individuals. The volunteers will fast overnight and drink sodium bicarbonate to neutralize stomach acid immediately before ingesting strain TW10722. The volunteers will be monitored in the ward for development of diarrhea and any other signs and symptoms from the gastrointestinal tract. All stools will be collected daily and cultured for the experimental ETEC strain. We will register time of evacuation, weight, and looseness of each stool that the volunteers pass. These data will be used to determine whether the volunteers got diarrhea, severity of diarrhea, incubation period, and maximum stool weight and volume during any 24-hour period. If they develop diarrhea (a single liquid stool totaling >200 ml, or >300 ml liquid total volume over 48 hr), they will be treated with the appropriate antibiotic drug to clear the infection within 24 hours of meeting any of these criteria or immediately if they show any signs of dehydration. In case of dehydration, oral rehydration solution, amounting to 150% of the weight of their stool, will be given at the start of diarrhea, and volunteers will be carefully monitored by a clinician (infectious disease specialist or anesthesiologist). Any volunteer who does not develop symptoms or signs within 120 hours will be treated with an appropriate antibiotic drug. For all participants, three consecutive stool samples must be verified as being negative for TW10722 prior to discharge from the hospital. Thus, the total duration of hospitalization is predicted to be ≤10 days.

The diarrhea/no diarrhea results from each new cohort will be used in a mathematical model (a beta-poisson model) designed to estimate the dose that is likely to give a 70% attack rate, and which is then given to the next cohort. The first cohort will receive 1×10^6 CFU, and each new cohort will at most receive one $\log_{10}$ higher dose than the previous group. If the optimal dose is found by using less than 30 volunteers, we wish to include the remaining volunteers

as a single group, all of whom will be given the estimated optimal dose. This will be done to ensure adequate statistical precision for the immunological analyses (objective 3 and 4) and we will also use the opportunity to test the quantitation method (objective 2).

Potential risks or discomforts

- Venepuncture or, if a volunteer should require intravenous (IV) rehydration (which was not required in any of the 30 volunteers in our previous study) starting an IV line may cause pain, bruising, lightheadedness, fainting, and very rarely infection. Very seldomly, bleeding may also occur from the site of venepuncture.
- The ETEC bacteria being used in this study are experimental agents. Based on previous studies with ETEC, the clinical effects that the volunteer are likely to have may include diarrhea, stomach pain/cramps, loss of appetite, headache, nausea, fever, fatigue, generalized joint and muscle aches and vomiting. Since these are experimental bacteria there is a possibility of side effects, including those that are not yet known. There is a risk of dehydration and electrolyte imbalances if diarrhea is severe. To keep the volunteer from getting dehydrated, he/she will be given fluids to drink, if clinical signs of some dehydration, we will give oral rehydration salts solution. If needed, i.e. if a violunteers shows signs or is at a risk of developing severe dehydration, fluids will be given intravenously. The volunteers will receive regular clinical examination by ward and study nurses, by study physicians and, when needed, by an anaesthesiologist.
- After ingesting the ETEC bacteria, the volunteer is likely to pass live ETEC bacteria in the stools for a few days.
- The antibiotics used to clear the ETEC infection, is usually well tolerated, but it could cause an allergic reaction (such as skin rashes, difficult breathing, or even a fatal reaction), diarrhea, nausea, and vomiting, abdominal pain/discomfort, headache, or restlessness or vaginal candidiasis (yeast infection) in women. If oral antibiotics cannot be taken, the volunteers will receive IV antibiotics.
- Although many experimental infection studies with ETEC have been undertaken during these last four decades and none have reported long term negative effects, we cannot rule out the possibility that the infection may lead to long-term sequelae, like post-infectious irritable bowel syndrome. From the three month follow-up of our previous study, there are no indications that any of our volunteers have had gastrointestinal problems after completing the study. See also external assessment with our comments in May 2010 (enclosed in REC application).

Sampling procedures

Other than for monitoring the health status of the volunteers during the study, their blood specimens will be used to accommodate objectives 3 and 4. Blood specimens will be collected five times from all volunteers: At the screening visit, within 24 hours of infection (day 0), and at days 7, 10, and 28. The stool specimens collected during the study may be stored and used for the development and testing of the strain- and ST-quantitation methods (objective 2) and for characterizing microbiota (objective 5). For monitoring secretory IgA in saliva, saliva will be collected by swabs on the screening day and on day 7, 10, and 28 (objective 3). For monitoring gut immune responses, we will perform an intestinal lavage on the day of screening and on day 10 (for objectives 3 and 4). For intestinal lavage, the volunteers will drink a solution that is used routinely to clean the colon prior to colonoscopy. It usually just stimulates the passage of watery stools ("intestinal lavage") until the subject stops drinking the solution. There may be minimal cramps. At least 100g of the lavage speimens will be collected for later antibody analysis. Intestinal lavage specimens are considered to be the gold standard for assessing gut immune responses.

Schedule of study events - ETECvac - Challenge Study TW10722

Procedure day	Screen	-1	0	1	2	4	5	6	7	10	28	84
Compliance range	"-60 to -1"	"-1 to 0"							"7-8"	"9-11"	"24 -30"	"70-98"
Outpatient visit	X									X	X	
Written informed consent	X											
History and patient examination	X											
Interim medical interview		X (2)	X	X	X	X	X	X	X	X	X	
Focused Physical examination		X (2)	X	X	X	X	X	X	X			
Blood draw	X	X (2)							X	X	X	
Screening lab tests (3)	X											
Hematology and Chemistry (4)	X									X		
HCG urine	X	X (2)									X	
Inpatient stay		X	X	X	X	X	X	X	X			
Challenge			X									
Stool collection/cultures	X (5)		X	X	X	X	X	X	X	X		
Stool weiging/Grading			X	X	X	X	X	X	X			
Stool bacteriology (strain detection)			X	X	X	X	X	X	X			
PBMCs for immunological assays		X (2)							X	X	X	
Saliva for immunology assays		X (2)							X	X	X	
Gastric lavage	X									X		
Antibiotic treatment (1)							X	X	X			
Discharge from inpatient phase									X			
3 month phone call												X
Maximal blood volume (ml)	60	90							80	90	80	

(1) Antbiotic treatment may be started sooner if the subject meets antibiotic tretament criteria
(2) Day "-1" procedures may be done on Day 0 before challange
(3) HIV, HBsAG, HCV, IgA
(4) CBC, WBC with differential, platelet count, serum chemistries, elctrolytes (Na, K, CO2), creatinine, s glucose, ALT
(5) Screening for intestinal pathogens

Confidentiality

To maintain confidentiality, coded numbers and initials will be used to identify the subject's laboratory specimens, source documents, Data Collection Forms, study reports etc. All study records will be maintained in a secure location. Clinical information will not be released without written permission from the subject, except as necessary for monitoring or auditing of the

patient. All collected specimens will be allocated a unique subject identification number, and only information on the age and sex of each subject will be available to the scientific research team.

2. Develop new methods for quantitating TW10722 and ST in stools

To be able to properly study the protective effect that an ST toxoid-based vaccine has on volunteers in a future Phase IIB trial, we will develop and establish new, sensitive methods to monitor the infection. For estimating the TW10722 load, we will use quantitative PCR that target one of the O115-antigen locus genes, which seems to be specific for a smaller number of ETEC. Since we normalize the results by dividing with total DNA amount of the analyzed sample, we expect that the TW10722 load estimate will be less affected by varying amounts of liquid in the analyzed stool sample, compared to the traditional method of colony counting. To estimate the ST load, we will develop a sensitive quantitative mass spectrometry method (stable isotope dilution-selected reaction monitoring [SID-SRM]). Stool specimens containing the ST to be measured will be spiked with a known amount of a slightly heavier, synthetic version of ST. A mass spectrometer, which is able to distinguish between the two, returns a ratio of normal to synthetic ST counts. The amount of ST in the original stool specimen can then be back-calculated by multiplying this ratio to the amount of synthetic ST added to the sample. Some of the stool specimens that the volunteers pass will be used for development and testing of these methods.

3. Characterize human immune responses induced by this experimental ETEC strain

Monitoring ETEC-specific salivary IgA antibodies can be used to measure the mucosal immune response. Mucosal homing antibody secreting cells (ASC) trafficing the blood can provide an indirect method of studying the mucosal response. The blood and lavage specimens will be used to evaluate the ETEC-specific antibody, T-cell, and B-cell responses induced after vaccination. We will thus obtain parallel gold standard readouts believed to reflect the gut response at various time intervals (0, 7, 10 and 28 days) after experimental infection to determine the time course of the response. Sera will be separated from clotted blood specimens and plasma will be separated from CPT heparinised blood, aliquoted and stored at -80°C or -20°C for use in appropriate immunological assays. Lymphocytes will be separated from CPT herparinised blood and used directly in the relevant immunological assays e.g. ELISPOT, ALS and for evaluation of the B and T cellular responses by e.g. flow cytometry (FACS) and cytokine production by multiplex ELISA and FACS. Antibodies in lymphocyte supernatant (ALS) will be stored and used to determine the frequency and magnitude of recent

mucosal immune responses to ETEC infection. We will develop and test sensitive flow cytometry multiplex bead and antigen-specific T-cell assays.
Excess lymphocytes will be stored in liquid nitrogen for later use in relevant immunological assays e.g. *in vitro* activation assays at the University of Bergen.

4. Screen for ETEC antigens responsible for inducing an immune response during experimental infection

The blood and lavage specimens will be used in screening studies against libraries of known and new protein antigens that TW10722 and other ETEC strains may be able to produce. By looking for difference in quantities of antigen-specific antibodies between before and after the infection with TW10722, we may identify antigens that are targeted by the immune system and which may be relevant for vaccine development.

5. Characterize changes in microbiota as a result of ETEC infection

Stool specimens will also be rapidly frozen in aliquoats and stored at -80°C. This will enable analysis of perturbations in the intestinal microbiota caused by ETEC infection. Such perturbations may modulate symptoms and severity of disease. Further funding will be sought to assess methods to to analyse whether certain microbiota compositions may be proftective against developing severe disease.

Project management, organization and cooperation

Collaborating institutions

- Centre for International Health, University of Bergen
- Section for Infectious Disease, Department Clinical Science, University of Bergen
- Unit for Infectious Diseases, Department of Medicine, Haukeland University Hospital, Bergen Department of Biomedicine, University of Bergen
- Bergen Clinical Vaccine Consortium, Haukeland University Hospital, Bergen
- Department of Anaesthesia and Intensive Care, Haukeland University HospitalDepartment of Clinical Research, University of Bergen.
- Department of Medical Microbiology, Haukeland University Hospital, Bergen
- Division of Infectious Disease Control, Norwegian Institute of Public Health, Oslo (NIPH)
  - Centre for Vaccine Develoment, University of Maryland, Baltimore, USA (CVD)

Professor Halvor Sommerfelt and researcher Hans Steinsland at CIH will provide the overall direction of this study. Postdoctoral fellow Kurt Hanevik will carry the medical responsability and coordinate clinical work.

Distribution of work

The Inoculum Preparation Laboratory of the Centre for Vaccine Development will produce the working cell bank of TW10722. The experimental infection work will be undertaken at the Unit for Infectious Diseases, HUS and Section for Infectious Disease, UiB, assisted by the Bergen Clinical Vaccine Consortium. Microbiological examinations will be undertaken at the Department of Medical Microbiology. Methods for quantitating ST and strain TW10722 will be developed at the Department of Biomedicine. Immunological analyses will be performed at the Section for Infectious Disease, UiB, and Norwegian Institute of Public Health, Oslo.

Study monitor

Ingunn H. Anundskås, Innovest AS

References

1. Walker RI, Steele D, Aguado T, Ad Hoc Technical Expert Committtee: Analysis of strategies to successfully vaccinate infants in developing countries against enterotoxigenic E. coli (ETEC) disease. Vaccine 2007, 25:2545–2566.
2. Kotloff KL, Blackwelder WC, Nasrin D, Nataro JP, Farag TH, van Eijk A, Adegbola RA, Alonso PL, Breiman RF, Faruque AS, Saha D, Sow SO, Sur D, Zaidi AK, Biswas K, Panchalingam S, Clemens JD, Cohen D, Glass RI, Mintz ED, Sommerfelt H, Levine MM. 2012. The Global Enteric Multicenter Study (GEMS) of diarrheal disease in infants and young children in developing countries: epidemiologic and clinical methods of the case/control study. Clinical infectious diseases: an official publication of the Infectious Diseases Society of America 55 Suppl 4:S232-245.
3. Levine MM, Ferreccio C, Prado V, Cayazzo M, Abrego P, Martinez J, Maggi L, Baldini MM, Martin W, Maneval D, et al. Epidemiologic studies of Escherichia coli diarrheal infections in a low socioeconomic level peri-urban community in Santiago, Chile. Am J Epidemiol. 1993; 138:849-69.
4. Valentiner-Branth P1, Steinsland H, Fischer TK, Perch M, Scheutz F, Dias F, Aaby P, Mølbak K, Sommerfelt H. Cohort study of Guinean children: incidence, pathogenicity, conferred protection, and attributable risk for enteropathogens during the first 2 years of life. J Clin Microbiol. 2003; 41:4238-45.
5. Taxt AM, Diaz Y, Bacle A, Grauffel C, Reuter N, Aasland R, Sommerfelt H, Puntervoll P. Characterization of immunological cross-reactivity between enterotoxigenic *Escherichia coli* heat-stable toxin and human guanylin and uroguanylin. Infect Immun. 2014;[Epub ahead of print].
6. Steinsland H, Valentiner-Branth P, Perch M, Dias F, Fischer TK, Aaby P, Mølbak K, Sommerfelt H. Enterotoxigenic Escherichia coli infections and diarrhea in a cohort of young children in Guinea-Bissau. J Infect Dis. 2002;186:1740-7.
7. Steinsland H, Lacher DW, Sommerfelt H, Whittam TS. Ancestral lineages of human enterotoxigenic Escherichia coli. J Clin Microbiol. 2010; 48:2916-24.

Relevant amendments to protocol August 2016

New ST-only challenge strain

ETEC bacterial strain used in the study was changed from TW10722 to TW11681.

Rationale: After performing a dose-escalation study in the fall of 2014, where we infected 3 groups of 3 volunteers with respectively $1x10^6$, $1x10^7$, and $1x10^8$ colony forming units of strain TW10722, few of the volunteers appeared to actually become colonized with the strain, and only one volunteer became ill and developed (severe) diarrhea.

For the next groups of volunteers, we wish to repeat dose escalation experiments with wild-type ETEC strain TW11681 (O19:H45; STh-CFA/I CS21), which belong to the epidemiologically relevant ETEC8 family. Results from phylogenetic and comparative genomics analyses of TW11681 and 19 other ETEC8 strains suggest that TW11681 is a good representative for ETEC8. Also, TW11681 is sensitive for the antibiotic we use (Ciprofloxacin), and does not seem to have any potential dangerous traits other than the ability to cause diarrhea.

Longevity of responses

The duration and strength of B and T cell memory responses in after ETEC infections are not well characterized. We therefore wish to do a follow up visit of volunteers of new volunteers challenged with the new TW11681 strain one year after the challenge, including a stool sample, a blood sample for immunological analyses of B and T cell responses. For the volunteers already challenged with TW10722 during autumn 2014 we would like to perform a two year follow up with these measurements.

Relevant amendments to protocol April 2017

ETEC bacterial strain used in the study was changed back to TW10722 and would be tested in higher doses. TW11681 seemed to predominantly cause non-diarrheal symptoms in nine volunteers, and therefore did not seem suitable as a challenge strain to measure a vaccine induced protection against secretory diarrhea.
